# What Determines That Older Adults Feel Younger Than They Are? Results From a Nationally Representative Study in Germany

**DOI:** 10.3389/fmed.2022.901420

**Published:** 2022-06-28

**Authors:** Konstantin G. Heimrich, Tino Prell, Aline Schönenberg

**Affiliations:** ^1^Department of Neurology, Jena University Hospital, Jena, Germany; ^2^Department of Geriatrics, Halle University Hospital, Halle, Germany

**Keywords:** aging, subjective age, depression, health, healthy aging, network, satisfaction with life

## Abstract

**Background:**

There is increasing evidence that subjective age is an important predictor of beneficial health outcomes besides chronological age. However, little is known about the factors associated with younger subjective age. This study aimed to identify which factors are predictive of feeling younger in old age. In this context, feeling younger was defined as an individual's perception of being younger than their current chronological age.

**Methods:**

Data from 4,665 community-dwelling older people were drawn from wave 7 (2020) of the German Aging Survey (DEAS), a nationally representative study in Germany. Network, mediation, and binomial logistic regression analyses were performed to reveal the associations between feeling younger and biopsychosocial factors.

**Results:**

A total of 4,039 participants reported feeling younger, while 626 did not. Older chronological age, engaging in sports more frequently, a better standard of living, a better state of health, higher satisfaction with life, more positive attitudes toward one's aging, and fewer depressive symptoms are associated with feeling younger in older people.

**Conclusion:**

The present study provides novel and consistent evidence regarding the association between feeling younger and biopsychosocial factors. Further research is needed to confirm these factors and identify how they can be adapted in potential intervention studies to generate the life and health circumstances that allow older people the benefit of feeling younger.

## Introduction

There is no uniformly specified age that defines an individual as old. Every person experiences the psychological and physiological processes during aging differently (1). In addition, subjective age is shaped by metacognitive beliefs about aging, including expectations and interpretations of individual experiences (2). Therefore, the chronological and subjective ages may differ significantly.

There is increasing evidence that, besides chronological age, subjective age is an important predictor of beneficial health outcomes. In particular, younger subjective age is associated with psychological wellbeing (3), younger estimated brain age assessed by regional gray matter volume (4), better cognitive functioning (5, 6), increased grip strength (7), faster walking speed (8), less frailty (9), a lower risk of cardiovascular diseases (10), hospitalization (11), and mortality (12). However, apart from these positive health effects, there is little knowledge about the factors contributing to younger subjective age, which may set the course for these beneficial outcomes. We hypothesized that several factors affect individuals' assessment of their subjective age.

The following section provides an overview of factors potentially relevant to subjective age. Although a person's chronological age and subjective age may differ significantly, recent evidence suggests an association between higher chronological age and lower subjective age (13). Besides age, sex differences may contribute to the assessment of subjective age, as there are sex-related differences in age stereotypes (14). However, studies investigating the relationship between an individual's sex and subjective age are inconsistent; therefore, this association needs to be further elucidated (15, 16).

Previous life circumstances and life events have a major impact on the quality of life of older adults (17). From the end of the Second World War until 1990, Germany was divided into the former Federal Republic of Germany in the western part and the former German Democratic Republic in the eastern part, with different social, economic, and healthcare systems. These differences may affect individual wellbeing, and there are economic and mortality disparities in both parts of Germany (18, 19). Accordingly, differences in the assignment of the place of residence to the east or west impact individuals' perceptions of age due to differences in the socioeconomic environment.

Furthermore, there are differences in subjective age based on education and perceived financial wellbeing, especially among older adults (20). In this regard, it seems plausible that receiving a pension or retirement benefit, which is often associated with a decrease in income, negatively affects subjective age. On the other hand, many low-income workers may find themselves forced to extend their working lives (21). In addition to objective or perceived income, retirement planning may also be influenced by subjective life expectancy (22) and, therefore, by individuals' perceptions of aging. The connection between retirement and subjective age has not yet been clarified. However, it is an expected normative of older age, indicating a certain stage in life, and may therefore influence subjective age.

Having a partner, child, family, or friend can provide social support to older adults. Social networks with high-quality social contacts are associated with subjective wellbeing in old age (23–25). Until now, little is known about the influence of social environment on subjective age. However, an immediate context is suspected and should be considered when evaluating subjective age (2).

Previous research suggests that physical health significantly impacts subjective age, as feeling younger is associated with better physical functioning (7, 8). Moreover, a large longitudinal study showed that higher physical activity is associated with a younger subjective age after 8–20 years (26). Accordingly, there seems to be a bidirectional relationship between feeling younger and physical activity. Besides reflecting on their physical abilities, individuals may also reflect on their mental health when evaluating their subjective age. Subjective age is also linked to depressive symptoms, loneliness, and satisfaction with life (27–30). Additionally, one can assume that the coronavirus pandemic may have a meaningful impact on individual aging views, resources, and coping strategies, and therefore, should be considered when evaluating subjective age.

Taken together, apart from the known beneficial health effects of a younger subjective age, there is little knowledge about the factors contributing to feeling younger and their interactions. Based on previous literature, several factors can be assumed to influence subjective age. Multifactorial analyses are necessary to consider the complex interactions between biology, psychology, and socio-environmental factors to understand their impact. There is an urgent need to obtain more evidence regarding biopsychosocial factors that promote the subjective assessment of feeling younger to reveal individual beneficial resources. This study aimed to identify predictors of feeling younger in older, community-dwelling people based on the results of a nationally representative study in Germany in 2020.

## Methods

### Study Design

Data are from the public release of wave 7 (2020) of the German Aging Survey (DEAS), provided by the Research Data Center of the German Center of Gerontology (DZA) (31). DEAS is a nationwide, representative, cross-sectional, and longitudinal survey of the German middle-aged and older population (minimum age 45 years), covering a wide range of topics and obtaining information on socioeconomic and demographic attributes, household composition, housing, family structure, social network, psychological resources, attitudes, and physical and mental health (31). The questionnaire, user manual, and codebook of wave 7 of the DEAS are publicly available *via* the website of the Research Data Center (https://www.dza.de). The microdata of DEAS are available free of charge to scientific researchers from the FDZ-DZA. Detailed information on the survey's design, content, and implementation is provided in the infas methodological report (32).

### Participants

Of the 4,823 respondents of wave 7 (2020) of the German Aging Survey (DEAS), 4,748 were community-dwelling individuals. We excluded 83 participants who did not indicate their perceived age, resulting in a sample of 4,665 participants.

### Outcome Parameter: Feeling Younger

This study defined *feeling younger* as the individual's perception of being younger than the current chronological age. Therefore, a binary variable indicating whether participants felt younger than their chronological age (*feeling_younger*) was generated using the chronological age (DEAS 2020 data variable *altervoll_20*) and perceived age (DEAS 2020 data variable *jp2*) of each participant. If the perceived age of the participant was lower than its chronological age, the participant was assigned to the group *feeling_younger* = “*1”*. If the perceived age of the participant was equal to or higher than their chronological age, the participant was assigned to the group *feeling_younger* = “*0”*.

### Independent Variables

Based on the previous literature and in accordance with the subject areas of wave 7 of the DEAS, several variables have been identified as potential factors pertaining to *feeling younger*. First, we considered demographic parameters including chronological *age* (metric, years), *sex* (male “1”, female “0”), receiving an old-age pension, disability pension benefit, or any *retirement* benefits (yes “1”, no “0”), and East-West-Assignment of place of *residence* (former Federal Republic of Germany “1”, former German Democratic Republic “0”). Level of *education* was considered with a three-stage dummy encoded variable (low “1”, medium “2”, high “3”). A low level of education refers to respondents without completed vocational qualifications and up to a maximum graduation degree, which qualifies for a professional qualification. A medium level of education refers to respondents with vocational qualifications or qualifications for a university or university of applied science entrance. A high level of education refers to respondents who have completed university studies (university or university of applied science).

Next, we included the parameters describing the social environment. The family structure was considered using the following variables: Having a spouse or steady *partner* (yes “1”, no “0”), living with the partner in the household (*living_partner*: yes “1”, no “0”), having a younger partner (*age_partner*: younger “1”, equal or older “0”), number of people in the *household*, number of *children*, and living with children in the household (*living_children*: yes “1”, no “0”). Respondents were asked to assess the quality of their current relationship with the partner (*relationship_partner*), family (*relationship_family*), and friends (*relationship_friends*) using a scale 5-point Likert scale ranging from 1 = very good to 5 = very bad. Contact with *neighbors* was additionally considered (very close “1” to no contact “5”).

Frequency of physical activity was assessed on a 6-point Likert scale ranging from 1 = daily to 6 = never for doing *sports* and going for *walks*. Additionally, respondents rated their *state_of* _*health* and their *standard_of_living* (very good “1”, good “2”, average “3”, bad “4”, very bad “5”). To assess the effect of the coronavirus pandemic, an infection of the respondent (*corona_infection*: infected “1”, not infected “0”) or of people in the personal environment (*corona_environment*: infected “1”, not infected “0”) were assessed. As well, participants were asked if the coronavirus crisis was perceived as a personal threat (*corona_threat*) on a 10-point Likert scale from “not at all a threat for me” to “extreme threat for me”.

Depressive mood was assessed using the German translation of the Center for Epidemiologic Studies Depression (CES-D) scale (short form, 10 items) with higher values indicating higher depressive symptoms. A score equal to or above 10 was considered depressed (33), and a dichotomous variable was determined (*depressive*: yes “1”, no “0”). Loneliness was assessed based on the 6-item De Jong Gierveld Loneliness Scale, with higher values indicating a higher level of loneliness (34). A mean value above 2.5 was considered as feeling lonely, and a dichotomous variable was determined (*loneliness*: yes “1”, no “0”). A five-item scale was used to assess life satisfaction (35). A mean value was generated, with higher values indicating a higher level of life satisfaction. A value above 3.0 was considered as satisfied with life, and a dichotomous variable was determined (*life_satisfaction*: yes “1”, no “0”). Finally, attitudes toward one's aging were assessed based on five items in accordance with the Philadelphia Geriatric Center Morale Scale (36). After recoding, high values indicated a positive attitude toward one's aging. A mean value above 2.5 was considered to represent a positive attitude toward one's aging, and a dichotomous variable was determined (*attitudes*: positive “1”, negative “0”).

### Statistical Analysis

Data were checked for normality using the Shapiro–Wilk test. Results were reported as the median and interquartile range (IQR) for non-normally distributed continuous variables or number (%) for categorical variables. For group comparisons, Mann–Whitney *U*-tests were performed for non-normally distributed ordinal data and chi-square tests for nominal data. The effect sizes of the Mann–Whitney *U*-test were given by the rank biserial correlation r_B_ and chi-square test by the Phi coefficient. Correlations between different clinical parameters were tested using Spearman's correlation r_s_. The effect sizes were considered low (|*r*_s_| = 0.1), moderate (|*r*_s_| = 0.3), or strong (|*r*_s_| = 0.5) (37). The level of statistical significance for all tests was set at *p* < 0.05 (two-tailed).

A network analysis was conducted to explore the association between *feeling younger* and the aforementioned biopsychosocial factors to depict their complex interplay. Therefore, the basic assumption is that analyzing the overall pattern of linkages between the variables provides a better explanation for interactions than considering separate correlations. A network estimates the relation between the variables directly without reducing the structure of the variables to their shared information. Each variable is an element of an interacting system. However, instead of presenting associations between all variables, which would lead to a confusing and unclear network, a regularization technique can be used. Thereby, interactions between two variables that are likely to be spurious are removed to allow easier interpretation of the network that focuses on significant relations. In this study, network characteristics and structure were assessed using the extended Bayesian information criterion (EBIC) (38, 39) with at least an absolute shrinkage and selection operator (LASSO) (40). Non-paranormal transformation of the non-normally distributed data was performed to achieve a normal distribution (npn). To ensure a more sensitive and specific network analysis, the tuning parameter of EBICglasso was set to 0.5. The nodes of the network display the variables, and they are positioned using the Fruchterman–Reingold algorithm based on the strength of the connections between nodes using pseudo-random numbers (41). The edges represent the correlations between nodes. The thickness of the edges corresponds to the strength of the correlation. Betweenness, closeness, and strength were determined as centrality measures, with relative values ranging from zero to one. Betweenness quantifies how often one node is on the shortest path between other nodes (42, 43). Closeness relies on the inverse sum of all distances from one node of interest to all other nodes and describes indirect connections (42, 43). Strength refers to the sum of the absolute input weights of that node and accordingly describes the direct connections of one node to other nodes (42–44). In general, higher centrality measures indicate that nodes are more central to the network. The stability of the centrality measures was estimated *via* a case-dropping bootstrap (number of bootstraps = 1,000) and quantified using the *CS coefficient*. The *CS coefficient* quantifies the proportion of cases that can be dropped to retain a correlation with the original centrality measure of higher than 0.7 in at least 95% of the samples (43). Above a cut-off of 0.5, the index can be considered stable (43). The accuracy of the network was estimated using a non-parametric bootstrapping procedure to assess the edge weight stability. Therefore, the edge weights' narrower 95% confidence intervals indicate a more trustworthy network (43). A bootstrapped difference test was also used to test whether the centrality measures of a node in the network were significantly different from each other node (43).

Based on the relationships within the explorative network plot, we identified potential mediators as nodes that were directly connected to two other nodes (outcome variable and potential predictor). We performed a simple mediation analysis to estimate the indirect and direct effects (*B*_ab_ and $B_{{\text{c}}^{\text{′}}}$) between potential nodes. The statistical significance of the effects was estimated using a bootstrapped procedure (number of bootstraps = 1,000).

Additionally, binomial logistic regression analyses with backward selection (likelihood ratio) were performed to identify predictors of *feeling younger*. Variables assessed using a Likert scale were analyzed ordinally. For the regression analyses, autocorrelation and multicollinearity were excluded (|*r*| < 0.8). Linearity was assessed using the Box–Tidwell procedure. Outliers were identified by calculating the standard deviation of the studentized residuals (SD > 3) and leverages (>0.2), and were subsequently excluded from further regression analyses.

SPSS (IBM SPSS Statistics, RRID:SCR_016479, version 27), JASP (JASP, RRID:SCR_015823, version 0.15), and Jamovi (jamovi, RRID:SCR_016142, version 2.2.5) were used for statistical analyses.

## Results

### Descriptive Analysis

The characteristics of the cohort are presented in Table 1. The majority of the entire cohort received pension or retirement benefits, lived in the western part of Germany, had two children, lived with a partner in the household, and assessed this conjugal relationship as good. The majority of participants were satisfied with their lives and did not feel lonely. The vast majority of participants were not infected with coronavirus and stated that there were no infections with coronavirus in their personal environment. The descriptive characteristics of the 4,039 participants (86.6%) who reported feeling younger (median reported age difference between participants' chronological age and their perceived age 9 years, IQR = 6–13 years, range 1–71 years) and 626 participants (13.4%) who reported not feeling younger (median reported age difference between participants' perceived age and their chronological age 1 year, IQR = 0–6 years, range 0–138 years) are shown in Table 1. The group comparisons are also shown in Table 1. The largest effect sizes of significant group differences were observed for state of health (*r*_B_ = 0.458; *p* < 0.001) and attitudes toward one's aging (Phi = 0.312; *p* < 0.001), with participants who were feeling younger rating their state of health more positive and reporting a more positive attitude toward their aging.

**Table 1:** Summary of variables stratified by *feeling younger*.

	**Total study cohort**	**Feeling younger**	**Not feeling younger**	***p***	***r***
	**(*N* = 4,665)**	**(*N* = 4,039)**	**(*N* = 626)**	**(2-sided)**	
Age (years), median (IQR)	69 (62–77)	70 (62–78)	67 (59–77)	<0.001^***^	−0.099
**Sex**				
0 Female	2,367 (50.7%)	2,062 (51.1%)	305 (48.7%)	0.278	0.016
1 Male	2,298 (49.3%)	1,977 (48.9%)	321 (51.3%)	0.278	−0.016
**Retirement**				
0 No	1,397 (30.3%)	1,172 (29.4%)	225 (36.8%)	<0.001^***^	−0.055
1 Yes	3,207 (69.7%)	2,821 (70.6%)	386 (63.2%)	<0.001^***^	0.055
**Residence**				
0 Former German democratic republic	1,430 (30.7%)	1,221 (30.2%)	209 (33.4%)	0.111	−0.023
1 Former federal republic of Germany	3,235 (69.3%)	2,818 (69.8%)	417 (66.6%)	0.111	0.023
**Education**				
1 Low	197 (4.2%)	166 (4.1%)	31 (5.0%)	0.325	−0.014
2 Medium	2,206 (47.3%)	1,870 (46.3%)	336 (53.8%)	0.001^**^	−0.051
3 High	2,261 (48.5%)	2,003 (49.6%)	258 (41.3%)	<0.001^***^	0.057
**Partner**				
0 No	1,064 (23.0%)	895 (22.6%)	151 (25.1%)	0.176	−0.020
1 Yes	3,509 (77.0%)	3,059 (77.4%)	450 (74.9%)	0.176	0.020
**Living_partner**				
0 No	194 (5.5%)	158 (5.2%)	36 (7.8%)	0.020^*^	−0.039
1 Yes	3,318 (94.5%)	2,894 (94.8%)	424 (92.2%)	0.020^*^	0.039
**Age_partner**				
0 Equal or older	1,698 (49.1%)	1,483 (49.3%)	215 (47.9%)	0.588	0.009
1 Younger	1,762 (50.9%)	1,528 (50.7%)	234 (52.1%)	0.588	−0.009
Household (*N*), median (IQR)	2 (2–2)	2 (2–2)	2 (2–2)	0.960	−0.001
Children (*N*), median (IQR)	2 (1–2)	2 (1–2)	2 (1–2)	0.261	−0.027
**Living_children**				
0 No	2,862 (81.5%)	2,503 (82.0%)	359 (78.0%)	0.041^*^	0.034
1 Yes	650 (18.5%)	549 (18.0%)	101 (22.0%)	0.041^*^	−0.034
Relationship_partner, median (IQR)	2 (1–2)	2 (1–2)	2 (1–2)	0.003^**^	0.080
1 Very good	1,255 (35.9%)	1,115 (36.6%)	140 (31.2%)	0.025^*^	0.038
2 Good	1,777 (50.9%)	1,547 (50.8%)	230 (51.2%)	0.868	−0.003
3 Average	397 (11.4%)	330 (10.8%)	67 (14.9%)	0.011^*^	−0.043
4 Bad	52 (1.5%)	45 (1.5%)	7 (1.6%)	0.894	−0.002
5 Very bad	13 (0.4%)	8 (0.3%)	5 (1.1%)	0.006^**^	−0.047
Relationship_family, median (IQR)	2 (2–2)	2 (2–2)	2 (2–3)	<0.001^***^	0.152
1 Very good	893 (19.6%)	805 (20.4%)	88 (14.6%)	0.001^**^	0.049
2 Good	2,558 (56.3%)	2,255 (57.2%)	303 (50.3%)	0.002^**^	0.047
3 Average	930 (20.5%)	764 (19.4%)	166 (27.6%)	<0.001^***^	−0.069
4 Bad	115 (2.5%)	91 (2.3%)	24 (4.0%)	0.014^*^	−0.036
5 Very bad	51 (1.1%)	30 (0.8%)	21 (3.5%)	<0.001^***^	−0.088
Relationship_friends, median (IQR)	2 (2–2)	2 (2–2)	2 (2–3)	<0.001^***^	0.142
1 Very good	556 (12.0%)	511 (12.7%)	45 (7.3%)	<0.001^***^	0.057
2 Good	2,977 (64.1%)	2,609 (64.8%)	368 (59.4%)	0.008^**^	0.039
3 Average	968 (20.8%)	798 (19.8%)	170 (27.4%)	<0.001^***^	−0.064
4 Bad	122 (2.6%)	94 (2.3%)	28 (4.5%)	0.002^**^	−0.046
5 Very bad	21 (0.5%)	12 (0.3%)	9 (1.5%)	<0.001^***^	−0.058
Neighbors, median (IQR)	3 (2–3)	3 (2–3)	3 (2–4)	<0.001^***^	0.087
1 Very close	112 (2.4%)	101 (2.5%)	11 (1.8%)	0.263	0.016
2 Close	1,271 (27.4%)	1,118 (27.9%)	153 (24.7%)	0.098	0.024
3 Not really close	2,242 (48.4%)	1,962 (48.9%)	280 (45.2%)	0.084	0.025
4 Only rare	919 (19.8%)	762 (19.0%)	157 (25.3%)	<0.001^***^	−0.054
5 No contact	89 (1.9%)	70 (1.7%)	19 (3.1%)	0.026^*^	−0.033
Sports, median (IQR)	3 (2–5)	3 (2–5)	4 (2–5)	<0.001^***^	0.252
1 Daily	557 (12.0%)	510 (12.7%)	47 (7.6%)	<0.001^***^	0.054
2 Several times a week	1,569 (33.9%)	1,436 (35.9%)	133 (21.4%)	<0.001^***^	0.104
3 Once a week	825 (17.8%)	707 (17.7%)	118 (19.0%)	0.419	−0.012
4 One to three times per month	283 (6.1%)	245 (6.1%)	38 (6.1%)	0.998	0.000
5 Less often	875 (18.9%)	724 (18.1%)	151 (24.3%)	<0.001^***^	−0.054
6 Never	514 (11.1%)	380 (9.5%)	134 (21.6%)	<0.001^***^	−0.131
Walks, median (IQR)	2 (2–4)	2 (2–4)	3 (2–5)	<0.001^***^	0.121
1 Daily	893 (19.3%)	787 (19.6%)	106 (17.3%)	0.168	0.020
2 Several times a week	1,732 (37.4%)	1,540 (38.4%)	192 (31.3%)	0.001^**^	0.050
3 Once a week	756 (16.3%)	659 (16.4%)	97 (15.8%)	0.693	0.006
4 One to three times per month	313 (6.8%)	275 (6.9%)	38 (6.2%)	0.540	0.009
5 Less often	757 (16.4%)	621 (15.5%)	136 (22.1%)	<0.001^***^	−0.061
6 Never	174 (3.8%)	129 (3.2%)	45 (7.3%)	<0.001^***^	−0.073
State_of_health, median (IQR)	2 (2–3)	2 (2–3)	3 (3–4)	<0.001^***^	0.458
1 Very good	344 (7.5%)	329 (8.2%)	15 (2.5%)	<0.001^***^	0.075
2 Good	2,255 (49.0%)	2,127 (53.3%)	128 (20.9%)	<0.001^***^	0.220
3 Average	1,628 (35.4%)	1,334 (33.4%)	294 (48.0%)	<0.001^***^	−0.104
4 Bad	342 (7.4%)	188 (4.7%)	154 (25.2%)	<0.001^***^	−0.265
5 Very bad	33 (0.7%)	12 (0.3%)	21 (3.4%)	<0.001^***^	−0.126
Standard_of_living, median (IQR)	2 (2–2)	2 (2–2)	2 (2–3)	<0.001^***^	0.275
1 Very good	1,032 (22.3%)	960 (23.9%)	72 (11.7%)	<0.001^***^	0.100
2 Good	2,454 (53.0%)	2,179 (54.3%)	275 (44.5%)	<0.001^***^	0.067
3 Average	990 (21.4%)	780 (19.4%)	210 (34.0%)	<0.001^***^	−0.121
4 Bad	127 (2.7%)	82 (2.0%)	45 (7.3%)	<0.001^***^	−0.109
5 Very bad	29 (0.6%)	13 (0.3%)	16 (2.6%)	<0.001^***^	−0.098
**Corona_infection**				
0 Not infected	4,351 (99.5%)	3,775 (99.5%)	576 (99.7%)	0.670	−0.006
1 Infected	20 (0.5%)	18 (0.5%)	2 (0.3%)	0.670	0.006
**Corona_environment**				
0 Not infected	4,144 (92.6%)	3,607 (92.8%)	537 (91.3%)	0.189	0.020
1 Infected	329 (7.4%)	278 (7.2%)	51 (8.7%)	0.189	−0.020
Corona_threat, median (IQR)	3 (2–5)	3 (2–5)	5 (3–6)	<0.001^***^	0.184
**Depressive**				
0 No	2,955 (64.5%)	2,745 (69.2%)	210 (34.4%)	<0.001^***^	0.247
1 Yes	1,624 (35.5%)	1,223 (30.8%)	401 (65.6%)	<0.001^***^	−0.247
**Loneliness**				
0 No	4,069 (90.0%)	3,580 (91.2%)	489 (82.3%)	<0.001^***^	0.100
1 Yes	450 (10.0%)	345 (8.8%)	105 (17.7%)	<0.001^***^	−0.100
**Life_satisfaction**				
0 No	611 (13.2%)	399 (10.0%)	212 (34.5%)	<0.001^***^	−0.246
1 Yes	4,002 (86.8%)	3,599 (90.0%)	403 (65.5%)	<0.001^***^	0.246
**Attitudes**				
0 Negative	1,209 (26.3%)	833 (20.9%)	376 (61.3%)	<0.001^***^	−0.312
1 Positive	3,384 (73.7%)	3,147 (79.1%)	237 (38.7%)	<0.001^***^	0.312

*Values are given as median and interquartile range unless otherwise indicated. Categorical parameters are given as absolute values and percentages. For group comparisons, Mann–Whitney U-tests were performed for non-normally distributed ordinal data and chi-square tests for nominal data*.

Significant group differences are indicated by ^*^

* *p < 0.05;*

** *p < 0.01;*

*** *p < 0.001*.

*The effect sizes (r) of the group differences were determined using the rank biserial correlation for the Mann–Whitney U-test and the Phi coefficient for the chi-square test*.

### Correlation Analysis

Within the entire cohort, univariate correlation analyses revealed a moderate correlation between feeling younger and a positive attitude toward one's aging (*r*_s_ = 0.312; *p* < 0.001), low correlations for engaging in sports more frequently (*r*_s_ = 0.153; *p* < 0.001), a better state of health (*r*_s_ = 0.295; *p* < 0.001), a higher standard of living (*r*_s_ = 0.178; *p* < 0.001), perceiving the coronavirus pandemic as less threatening (*r*_s_ = 0.110; *p* < 0.001), fewer depressive symptoms (*r*_s_ = 0.247; *p* < 0.001), feeling less lonely (*r*_s_ = 0.100; *p* < 0.001), and higher satisfaction with life (*r*_s_ = 0.246; *p* < 0.001) (detailed in Supplementary Table S1).

### Network Analysis

**Figure 1:**
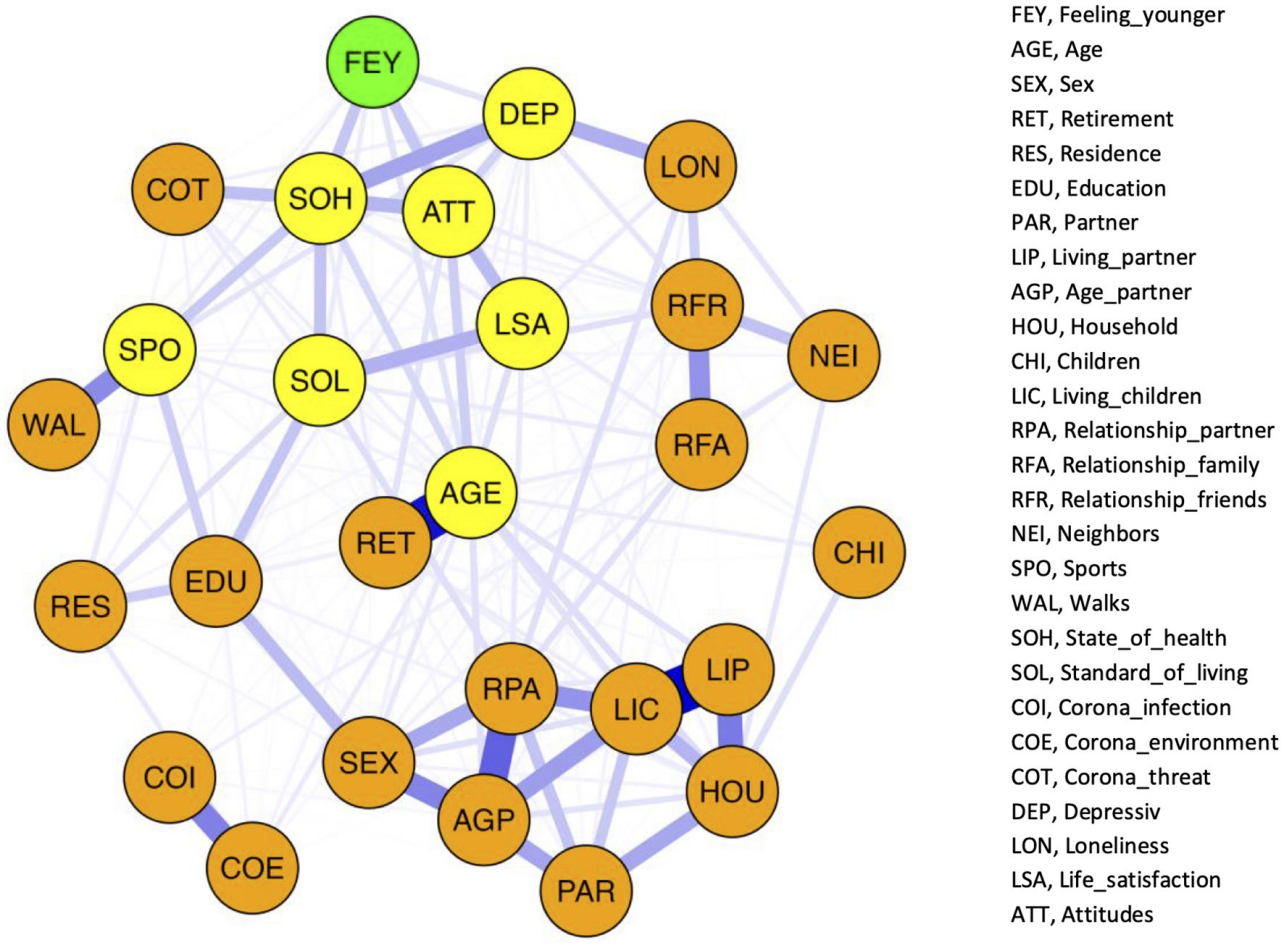
Network structure of the total study population. The nodes display the variables and the edges represent correlations between the nodes. The thickness of the edges corresponds to the strength of the correlation. Green: *Feeling_young*er (FEY), Yellow: variable directly associated with FEY (AGE, *age*; SPO, *sports*; SOH, *state_of_health*; SOL, *standard_of_living*; DEP, *depressive*; LSA, *life_satisfaction*; ATT, *attitudes*). Orange: variables of the network not directly associated with FEY.

**Figure 2:**
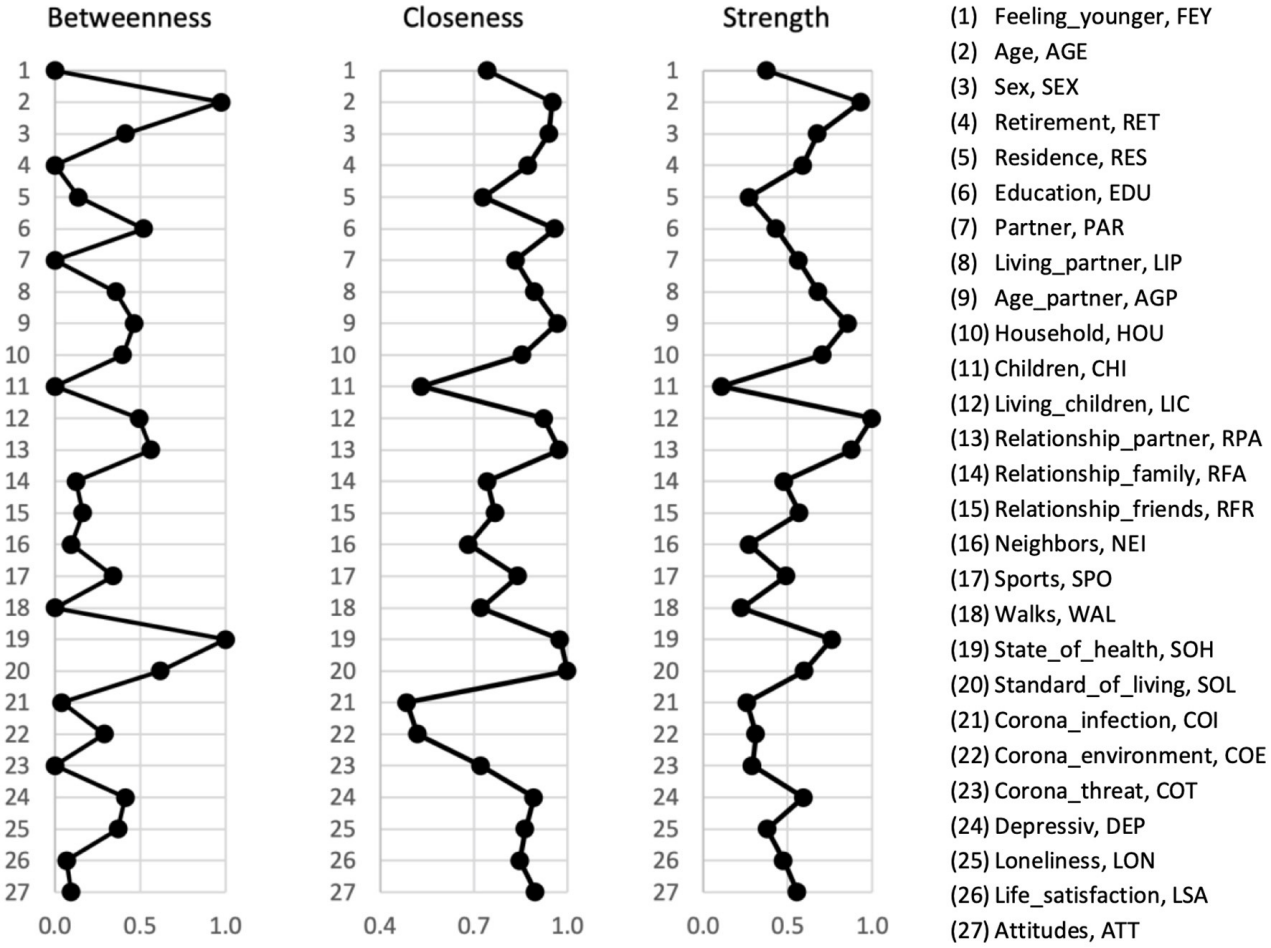
Node centrality of the total study population. Centrality indices for betweenness, closeness, and strength are given in relative values. A higher centrality measures indicate that the node is more central to the network.

A network plot of the 27 variables is shown in Figure 1. The nodes display the variables, and the thickness of the edges represents the strength of the correlations between the nodes. Network analysis revealed a well-connected network without isolated nodes; 210 out of 351 edges were estimated to be above zero. For each variable, the centrality indices for betweenness, closeness, and strength of the total study population are shown in Figure 2 (and tabulated in Supplementary Table S2). Additionally, detailed edge weights are shown in Supplementary Table S3. The highest betweenness was determined for the participants' state of health (SOH). This node substantially impacts the network because it is most often located on the shortest connections between pairs of other nodes. Accordingly, participants' state of health (SOH) may affect the communication between many pairs of nodes. Standard of living (SOL) had the highest closeness centrality measure. The distances from this node to all other nodes were the shortest. Therefore, standard of living (SOL) showed the strongest indirect connections within the network. Moreover, it can be noted that living with children (LIC) had the highest strength centrality measure. This means that this node has the highest input weights from the other directly connected nodes. Accordingly, living with children (LIC) has a considerable influence on several connected factors.

On a global level, the network can be visually divided into four domains, which are shown in Supplementary Figure S1: items describing household composition (SEX, *sex*; PAR, *partner*; AGP, *age_partner*; RPA, *relationship_partner*; LIP, *living_partner*; LIC, *living_children*; CHI, *children*; HOU, *household*), items describing social contacts (LON, *loneliness*; RFA, *relationship_family*; RFR, *relationship_friends*; NEI, *neighbors*), items describing wellbeing (FEY, *feeling_younger*; SOH, *state_of_health*; ATT, *attitudes*; DEP, *depressive*; COT, *corona_threat*; SOL, *standard_of_living*; LSA, *life_satisfaction*; SPO, *sports*; WAL, *walks*), and items related to a coronavirus infection (COI, *corona_infection*; COE, *corona_environment*). Four items could not be categorized into one of these domains (AGE, *age*; RET, *retirement*; EDU, *education*; and RES, *residence*). However, these items are not isolated. They pass the information of the household composition domain and the coronavirus infection domain to the wellbeing domain. In general, these four domains are not strictly separated. There are various cross-domain associations. In particular, there is a strong connection between social contact and wellbeing domains *via* loneliness (LON). Loneliness has a direct effect on feeling younger (FEY). However, the indirect effect mediated by depression (DEP) predominated (*B*_ab_ = −0.069, *p* < 0.001, 61.3%; $B_{{\text{c}}^{\text{′}}}$ = −0.044, *p* = 0.037, 38.7%).

Feeling younger (FEY) was not the most centrally located factor within the network, as specified by the centrality measures betweenness, closeness, and strength. The betweenness of feeling younger is very low (0.000). Accordingly, feeling younger does not affect communication between pairs of other nodes. However, for feeling younger, a strong closeness centrality measure was determined (0.744), referring to the distances to all other nodes and, therefore, the indirect connections within the network. Based on the moderate strength centrality measure (0.377), feeling younger was directly associated with several network factors. In this regard, our network structure revealed direct associations between feeling younger (FEY) and the chronological age of the participants (AGE), frequency of engaging in sports (SPO), state of health (SOH), standard of living (SOL), depressive symptoms (DEP), participants' satisfaction with life (LSA), and attitudes toward one's aging (ATT). Therefore, the edge weights between FEY and both ATT and SOH were high, corresponding to strong correlations. If we look at these connections, it becomes apparent that SOH seems to mediate the association between SOL and FEY, as well as the association between SPO and FEY. Subsequent mediation analysis revealed that the association between SPO and FEY was partially mediated by SOH (*B*_ab_ = −0.017, *p* < 0.001, 52.1%; $B_{{\text{c}}^{\text{′}}}$ = −0.016, *p* < 0.001, 47.9%). Additionally, the state of health partially mediated the effect of standard of living on feeling younger (*B*_ab_ = −0.040, *p* < 0.001, 47.0%; $B_{{\text{c}}^{\text{′}}}$ = −0.045, *p* < 0.001, 53.0%). In addition, attitudes toward one's aging partially mediated the association between life satisfaction and feeling younger (*B*_ab_ = 0.093, *p* < 0.001, 37.2%; $B_{{\text{c}}^{\text{′}}}$ = 0.157, *p* < 0.001, 62.8%).

The results of the network accuracy and stability analyses are shown in Supplementary Figures S2–S4. The case-dropping bootstrapping procedure showed that the centrality measures of betweenness [CS (cor = 0.7) = 0.66], closeness [CS (cor = 0.7) > 0.75], and strength [CS (cor = 0.7) > 0.75] remained highly stable. The non-parametric bootstrapping procedure revealed that the identified edge weights were accurate and that the centrality indices of the nodes were significantly different from each other.

### Regression Analysis

Spearman correlations between independent variables were high (*r* > 0.8), indicating that multicollinearity was a confounding factor in the analysis (*education_2* and *education_3*: *r* = −0.916, *p* < 0.001; *partner* and *living_partner*: *r* = 0.899, *p* < 0.001; *household* and *living_children*: *r* = 0.852, *p* < 0.001). Therefore, *education_2, partner*, and *household* were excluded from the regression analysis.

We conducted a logistic regression analysis within the study population of community-dwelling people [χ^2^_(11)_ = 353.20, *p* < 0.001, Nagelkerk's *R*^2^ = 0.241] to understand the predictors of feeling younger. Four participants were identified as outliers and excluded from further analysis. Feeling younger was associated with older age [Exp(B) = 1.025; *p* = 0.012] and fewer depressive symptoms [Exp(B) = 1.546; *p* = 0.007]. Additionally, if participants are doing sports less frequently [Exp(B) = 0.908; *p* = 0.026], have a worse state of health [Exp(B) = 0.533; *p* < 0.000], a worse standard of living [Exp(B) = 0.811; *p* = 0.026], are less satisfied with their life [Exp(B) = 0.632; *p* = 0.016], and report a negative attitude toward their aging [Exp(B) = 0.360; *p* < 0.001], they are less likely to feel younger. For detailed information, steps 1 and 15 of the regression are shown in Supplementary Table S4.

## Discussion

Subjective age is an important predictor of positive health outcomes (3–12). In a representative sample of 4,665 older community-dwelling people in Germany, the majority reported feeling younger than their chronological age. We identified several biopsychosocial factors associated with feeling younger.

Both network analysis and regression analysis revealed that older chronological age, engaging in sports more frequently, a better standard of living, a better state of health, higher satisfaction with life, more positive attitudes toward one's aging, and fewer depressive symptoms are associated with feeling younger in older people. However, as suggested by network analysis and confirmed by mediation analysis, in particular state of health, attitudes toward one's aging and depression have a considerable direct effect on subjective age.

Our study revealed that older chronological age is associated with feeling younger. This at first glance paradoxical finding can be interpreted in line with previous research, showing an association between higher chronological age and lower subjective age (13). Participants who were feeling younger reported a subjective age that was on average 9 years younger than their chronological age. Feeling younger contributes to mentally distancing oneself from an age group associated with a decline in functioning, which might allow older people to maintain a more positive view of themselves (46). People often report a younger subjective age when comparing themselves to people with worse health, or when they conclude that despite their increasing age, they do not change significantly (16, 47, 48). Accordingly, ageism, which refers to stereotyping individuals or groups based on age, contributes significantly to the assessment of subjective age. Furthermore, ageism is known to affect people's health negatively (49–51). It can be assumed that the coronavirus pandemic and the focus on older vulnerable persons have aggravated ageism (52). Ageism is negatively related to subjective health and life satisfaction after the onset of the pandemic, and younger subjective age may buffer this negative effect (53). In this context, feeling young may be seen as a beneficial coping process (54). Our data were derived from the German Aging Survey conducted from June to July 2020 after the first wave of the coronavirus pandemic. In our study, participants who felt younger perceived the coronavirus crisis as less threatening than participants who did not feel younger. However, most of the participants did not perceive the coronavirus pandemic as a personal threat.

The present study revealed that feeling younger was associated with engaging in sports more frequently, which was partially mediated by a better state of health. This is in line with previous research showing an association between feeling younger and faster walking speed (8) as well as overall better physical functioning (7, 26). Additionally, this finding corresponds to the fact that physical activity enables healthy aging and reduces mortality (45, 55, 56). Therefore, it should be noted that physical activity improves both physical and mental health (56), which may improve the self-perception of aging.

In line with previous research, our study confirms that feeling younger is also associated with fewer depressive symptoms (27, 57). This finding may be explained by the association of late-life resources and strategies for coping with depressive symptoms (58). Previous research has shown that affective mental health factors, such as depression impact subjective aging views (59). Likewise, life satisfaction, self-esteem, and purpose in life are known predictors of lower depressive symptoms (60). Accordingly, increasing life satisfaction can potentially prevent or alleviate depressive symptoms. Additionally, independent of depressive symptoms, higher satisfaction with life itself is also associated with feeling younger, as the present study was able to show. However, it should be noted that less favorable individual aging attitudes contribute to the association between an older subjective age and lower life satisfaction (61, 62). Our study revealed that attitudes toward one's aging partially mediate the relationship between feeling younger and life satisfaction. Higher satisfaction with life may correspond to more positive aging attitudes, which reduce age-related attributions of changes. More positive attitudes toward one's aging improve the subjective experience of aging, and, accordingly, it is more likely that someone reports feeling younger. These results align with a previous longitudinal study showing that an increase in positive attitudes toward one's aging results in a relative decrease in subjective age (63). In summary, our results underline the importance of both mental and physical health in terms of subjective age. The determination of the state of health can be seen as an aggregated assessment of the mental and physical health domains. State of health is one of the most central variables within the network structure of our study, according to the betweenness centrality measure. Therefore, we were able to reveal that feeling younger was associated with an overall better state of health.

In addition to mental and physical health, social environment is an important contributor to subjective age. Our regression analysis found that a higher standard of living was associated with feeling younger. Moreover, our network and mediation analyses revealed that this connection is partially mediated by a better state of health. This is in line with previous research showing that perceived income affects self-rated health (64, 65).

To the best of our knowledge, this is the first study applying network analysis to assess the impact of complex interacting biopsychosocial factors, that determine feeling younger in older age. However, our study has several limitations. First, our study did not include people living in nursing homes because of the small number of respondents in the questionnaire. Therefore, the results cannot be generalized to older adults living in residential care. Second, we cannot exclude positive selection errors. It is plausible that participants who answered the questionnaire may have had better physical and mental capabilities. In addition, there is an association between cognitive deficits and older subjective age (6). Therefore, it is probable that our available data underestimate the proportion of people feeling older than their chronological age. Third, dichotomizing feeling younger to assess subjective age risks losing information. However, it is an established method for determining subjective age (6, 8, 12, 26, 54). Fourth, as independent variables we only considered variables of wave 7 of the DEAS. Accordingly, there are other possible influential factors on subjective age that could not be taken into account within this study. Fifth, because of the cross-sectional design, no statement can be made on the effect of time-dependent variables, including short-term changes as well as long-term influences. We applied network analysis to cross-sectional data as an initial step to gain an overview of the many variables potentially related to feeling younger in advanced age, however, it would be beneficial to follow up this study with analysis on longitudinal data. Finally, because of the cross-sectional design, no causal effects could be determined. It is not clear whether the aforementioned factors predominantly lead to a younger subjective age or if they are the main consequences of a younger subjective age.

Taken together, the present study provides novel evidence regarding the association between feeling younger and several biopsychosocial factors. Our analyses revealed that an older chronological age, engaging in sports more frequently, a better standard of living, a better state of health, higher satisfaction with life, more positive attitudes toward one's aging, and fewer depressive symptoms are associated with feeling younger in older people. Further research is needed to confirm these factors and identify how they can be adapted in potential intervention studies to generate the life and health circumstances that allow older people the benefit of feeling younger.

## Data Availability Statement

Publicly available datasets were analyzed in this study. This data can be found here: https://www.dza.de.

## Ethics Statement

Ethical review and approval was not required for the study on human participants in accordance with the local legislation and institutional requirements. Written informed consent for participation was not required for this study in accordance with the national legislation and the institutional requirements.

## Author Contributions

KH and TP: study concept and design. KH, AS, and TP: statistical analysis and interpretation of data. KH: first draft of the manuscript. AS and TP: critical revision of the manuscript. Final approval was obtained from all authors.

## Funding

Funding to KH was provided by the Deutsche Forschungsgemeinschaft (DFG, German Research Foundation) in the Clinician Scientist-Program OrganAge, Funding Number 413668513 and the Interdisciplinary Center of Clinical Research of the Medical Faculty of Jena.

## Conflict of Interest

The authors declare that the research was conducted in the absence of any commercial or financial relationships that could be construed as a potential conflict of interest.

## Publisher's Note

All claims expressed in this article are solely those of the authors and do not necessarily represent those of their affiliated organizations, or those of the publisher, the editors and the reviewers. Any product that may be evaluated in this article, or claim that may be made by its manufacturer, is not guaranteed or endorsed by the publisher.

## References

[1] Kotter-Grühn D Kornadt AE Stephan Y. Looking beyond chronological age: current knowledge and future directions in the study of subjective age. Gerontology. (2015) 62:86–93. 10.1159/000438671 26302678

[2] Hughes ML Touron DR. Aging in context: incorporating everyday experiences into the study of subjective age. Front Psychiatry. (2021) 12:633234. 10.3389/fpsyt.2021.633234 33897492 PMC8062800

[3] Weiss D Sassenberg K Freund AM. When feeling different pays off: how older adults can counteract negative age-related information. Psychol Aging. (2013) 28:1140–6. 10.1037/a0033811 23957227

[4] Kwak S Kim H Chey J Youm Y. Feeling how old i am: subjective age is associated with estimated brain age. Front Aging Neurosci. (2018) 10:168. 10.3389/fnagi.2018.00168 29930506 PMC5999722

[5] Stephan Y Caudroit J Jaconelli A Terracciano A. Subjective age and cognitive functioning: a 10-year prospective study. Am J Geriatr Psychiatry. (2014) 22:1180–7. 10.1016/j.jagp.2013.03.007 23871114

[6] Stephan Y Sutin AR Luchetti M Terracciano A. Feeling older and the development of cognitive impairment and dementia. J Gerontol B Psychol Sci Soc Sci. (2017) 72:966–73. 10.1093/geronb/gbw085 27436103 PMC5927095

[7] Stephan Y Chalabaev A Kotter-Grühn D Jaconelli A. “Feeling younger, being stronger”: an experimental study of subjective age and physical functioning among older adults. J Gerontol B Psychol Sci Soc Sci. (2013) 68:1–7. 10.1093/geronb/gbs037 22492113

[8] Stephan Y Sutin AR Terracciano A. “Feeling younger, walking faster”: subjective age and walking speed in older adults. Age. (2015) 37:86. 10.1007/s11357-015-9830-9 26296609 PMC5005834

[9] Li Y Liu M Miyawaki CE Sun X Hou T Tang S. Bidirectional relationship between subjective age and frailty: a prospective cohort study. BMC Geriatr. (2021) 21:395. 10.1186/s12877-021-02344-1 34187378 PMC8244193

[10] Stephan Y Sutin AR Wurm S Terracciano A. Subjective aging and incident cardiovascular disease. J Gerontol B Psychol Sci Soc Sci. (2021) 76:910–9. 10.1093/geronb/gbaa106 32857131 PMC8063671

[11] StephanYSutinARTerraccianoA. Feeling older and risk of hospitalization: evidence from three longitudinal cohorts. Health Psychol. (2016) 35:634–7. 10.1037/hea000033526867044

[12] Stephan Y Sutin AR Terracciano A. Subjective age and mortality in three longitudinal samples. Psychosom Med. (2018) 80:659–64. 10.1097/PSY.0000000000000613 29864106 PMC6345273

[13] Pinquart M Wahl HW. Subjective age from childhood to advanced old age: a meta-analysis. Psychol Aging. (2021) 36:394–406. 10.1037/pag0000600 33829847

[14] Kornadt AE Voss P Rothermund K. Multiple standards of aging: gender-specific age stereotypes in different life domains. Eur J Ageing. (2013) 10:335–44. 10.1007/s10433-013-0281-9 28804307 PMC5549213

[15] Pinquart M Sörensen S. Gender differences in self-concept and psychological well-being in old age: a meta-analysis. J Gerontol B Psychol Sci Soc Sci. (2001) 56:P195–213. 10.1093/geronb/56.4.P195 11445606

[16] Sabatini S Ukoumunne OC Ballard C Collins R Kim S Corbett A. What does feeling younger or older than one's chronological age mean to men and women? qualitative and quantitative findings from the protect study. Psychol Health. (2021) 36:1–24. 10.1080/08870446.2021.1960989 34353194

[17] Enwo OO Player E Steel N Ford JA. The impact of life events on later life: a latent class analysis of the english longitudinal study of ageing. J Public Health. (2021) 43:e180–e7. 10.1093/pubmed/fdaa002 32157284

[18] van Raalte AA Klüsener S Oksuzyan A Grigoriev P. Declining regional disparities in mortality in the context of persisting large inequalities in economic conditions: the case of Germany. Int J Epidemiol. (2020) 49:486–96. 10.1093/ije/dyz265 31977053 PMC7266541

[19] Grigoriev P Pechholdová M Mühlichen M Scholz RD Klüsener S. 30 years of German unification: achievements and remaining differences in mortality trends by age and cause of death. Bundesgesundheitsblatt Gesundheitsforschung Gesundheitsschutz. (2021) 64:481–90. 10.1007/s00103-021-03299-9 33765247 PMC8060242

[20] Barrett AE. Socioeconomic status and age identity: the role of dimensions of health in the subjective construction of age. J Gerontol B Psychol Sci Soc Sci. (2003) 58:S101–9. 10.1093/geronb/58.2.S101 12646599

[21] Hasselhorn HM Ebener M Vratzias A. Household income and retirement perspective among older workers in Germany-findings from the lida cohort study. J Occup Health. (2020) 62:e12130. 10.1002/1348-9585.12130 32515884 PMC7221420

[22] Solinge H Henken K. Living longer, working longer? The impact of subjective life expectancy on retirement intentions and behavior. Eur J Public Health. (2010) 20:47–51. 10.1093/eurpub/ckp118 19822568

[23] Becker C Kirchmaier I Trautmann ST. Marriage, parenthood and social network: subjective well-being and mental health in old age. PLoS ONE. (2019) 14:e0218704. 10.1371/journal.pone.0218704 31339896 PMC6656342

[24] Pinquart M Sörensen S. Influences of socioeconomic status, social network, and competence on subjective well-being in later life: a meta-analysis. Psychol Aging. (2000) 15:187–224. 10.1037/0882-7974.15.2.187 10879576

[25] Tomini F Tomini SM Groot W. Understanding the value of social networks in life satisfaction of elderly people: a comparative study of 16 european countries using share data. BMC Geriatr. (2016) 16:203. 10.1186/s12877-016-0362-7 27905902 PMC5134265

[26] Stephan Y Sutin AR Terracciano A. Physical activity and subjective age across adulthood in four samples. Eur J Ageing. (2020) 17:469–76. 10.1007/s10433-019-00537-7 33381000 PMC7752936

[27] Alonso Debreczeni F Bailey PE. A systematic review and meta-analysis of subjective age and the association with cognition, subjective well-being, and depression. J Gerontol B Psychol Sci Soc Sci. (2021) 76:471–82. 10.1093/geronb/gbaa069 32453828

[28] Vitman Schorr A Yehuda I Tamir S. Loneliness, malnutrition and change in subjective age among older adults during Covid-19 pandemic. Int J Environ Res Public Health. (2020) 18:106. 10.3390/ijerph18010106 33375219 PMC7796152

[29] Ayalon L Palgi Y Avidor S Bodner E. Accelerated increase and decrease in subjective age as a function of changes in loneliness and objective social indicators over a four-year period: results from the health and retirement study. Aging Ment Health. (2016) 20:743–51. 10.1080/13607863.2015.1035696 25925282

[30] Brothers A Miche M Wahl H-W Diehl M. Examination of associations among three distinct subjective aging constructs and their relevance for predicting developmental correlates. J Gerontol B Psychol Sci Soc Sci. (2017) 72:547–60. 10.1093/geronb/gbv085 26430165 PMC5927160

[31] Klaus D Engstler H Mahne K Wolff JK Simonson J Wurm S. Cohort profile: the German ageing survey (Deas). Int J Epidemiol. (2017) 46:1105-g. 10.1093/ije/dyw326 28180273 PMC5837219

[32] Infas IfaSG. Methodenbericht Deutscher Alterssurvey (Deas): Schriftliche Panelbefragung. Bonn: Institut für angewandte Sozialwissenschaft GmbH (2020).

[33] Andresen EM Malmgren JA Carter WB Patrick DL. Screening for depression in well older adults: evaluation of a short form of the Ces-D (center for epidemiologic studies depression scale). Am J Prev Med. (1994) 10:77–84. 10.1016/S0749-3797(18)30622-6 8037935

[34] Gierveld JDJ Tilburg TV. A 6-item scale for overall, emotional, and social loneliness: confirmatory tests on survey data. Res Aging. (2006) 28:582–98. 10.1177/0164027506289723

[35] Pavot W Diener E. Review of the satisfaction with life scale. Psychol Assess. (1993) 5:164–72. 10.1037/1040-3590.5.2.164

[36] Lawton MP. The Philadelphia geriatric center morale scale: a revision. J Gerontol. (1975) 30:85–9. 10.1093/geronj/30.1.85 1109399

[37] Cohen J. Statistical Power Analysis for the Behavioral Sciences. 2nd ed. Hillsdale, NJ: Erlbaum (1988).

[38] Foygel R Drton M. Extended bayesian information criteria for Gaussian graphical models. In: Proceedings of the 23rd International Conference on Neural Information Processing Systems, Vol. 1. Vancouver, BC (2010). p. 604–12.

[39] Chen J Chen Z. Extended bayesian information critera for model selection with large model spaces. Biometrika. (2008) 95:759–71. 10.1093/biomet/asn034

[40] Friedman J Hastie T Tibshirani R. Sparse inverse covariance estimation with the graphical lasso. Biostatistics. (2008) 9:432–41. 10.1093/biostatistics/kxm045 18079126 PMC3019769

[41] Fruchterman TM Reingold EM. Graph drawing by force-directed placement. Software Pract Exp. (1991) 21:1129–64. 10.1002/spe.4380211102

[42] Epskamp S Fried EI. A tutorial on regularized partial correlation networks. Psychol Methods. (2018) 23:617–34. 10.1037/met0000167 29595293

[43] Epskamp S Borsboom D Fried EI. Estimating psychological networks and their accuracy: a tutorial paper. Behav Res Methods. (2018) 50:195–212. 10.3758/s13428-017-0862-1 28342071 PMC5809547

[44] Opsahl T Agneessens F Skvoretz J. Node centrality in weighted networks: generalizing degree and shortest paths. Soc Networks. (2010) 32:245–51. 10.1016/j.socnet.2010.03.006

[45] Hupin D Roche F Gremeaux V Chatard JC Oriol M Gaspoz JM. Even a low-dose of moderate-to-vigorous physical activity reduces mortality by 22% in adults aged ≥60 years: a systematic review and meta-analysis. Br J Sports Med. (2015) 49:1262–7. 10.1136/bjsports-2014-094306 26238869

[46] Weiss D Freund AM. Still young at heart: negative age-related information motivates distancing from same-aged people. Psychol Aging. (2012) 27:173–80. 10.1037/a0024819 21823797

[47] Rickabaugh CA Tomlinson-Keasey C. Social and temporal comparisons in adjustment to aging. Basic Appl Soc Psych. (1997) 19:307–28. 10.1207/s15324834basp1903_3

[48] Ferring D. Hoffmann M. “Still the same and better off than others?” Social and temporal comparisons in old age. Eur J Ageing. (2007) 4:23–34. 10.1007/s10433-007-0045-5 28794769 PMC5546364

[49] Officer A Schneiders ML Wu D Nash P Thiyagarajan JA Beard JR. Valuing older people: time for a global campaign to combat ageism. Bull World Health Organ. (2016) 94:710-a. 10.2471/BLT.16.184960 27843156 PMC5043217

[50] Lamont RA Swift HJ Abrams D. A review and meta-analysis of age-based stereotype threat: negative stereotypes, not facts, do the damage. Psychol Aging. (2015) 30:180–93. 10.1037/a0038586 25621742 PMC4360754

[51] Meisner BA. A meta-analysis of positive and negative age stereotype priming effects on behavior among older adults. J Gerontol B Psychol Sci Soc Sci. (2012) 67:13–7. 10.1093/geronb/gbr062 21746872

[52] Fraser S Lagacé M Bongué B Ndeye N Guyot J Bechard L. Ageism and Covid-19: what does our society's response say about us? Age Ageing. (2020) 49:692–5. 10.1093/ageing/afaa097 32377666 PMC7239227

[53] Kornadt AE Albert I Hoffmann M Murdock E Nell J. Ageism and older people's health and well-being during the Covid-19-pandemic: the moderating role of subjective aging. Eur J Ageing. (2021) 18:1–12. 10.1007/s10433-021-00624-8 33948107 PMC8085090

[54] Terracciano A Stephan Y Aschwanden D Lee JH Sesker AA Strickhouser JE. Changes in subjective age during Covid-19. Gerontologist. (2021) 61:13–22. 10.1093/geront/gnaa104 32766780 PMC7454556

[55] Chodzko-Zajko WJ Proctor DN Fiatarone Singh MA Minson CT Nigg CR Salem GJ. American college of sports medicine position stand. Exercise and physical activity for older adults. Med Sci Sports Exerc. (2009) 41:1510–30. 10.1249/MSS.0b013e3181a0c95c 19516148

[56] Cunningham C O' Sullivan R Caserotti P Tully MA. Consequences of physical inactivity in older adults: a systematic review of reviews and meta-analyses scand. J Med Sci Sports. (2020) 30:816–27. 10.1111/sms.13616 32020713

[57] Keyes CL Westerhof GJ. Chronological and subjective age differences in flourishing mental health and major depressive episode. Aging Ment Health. (2012) 16:67–74. 10.1080/13607863.2011.596811 21780972

[58] Bjørkløf GH Engedal K Selbæk G Kouwenhoven SE Helvik AS. Coping and depression in old age: a literature review. Dement Geriatr Cogn Disord. (2013) 35:121–54. 10.1159/000346633 23392253

[59] Schönstein A Dallmeier D Denkinger M Rothenbacher D Klenk J Bahrmann A. Health and subjective views on aging: longitudinal findings from the actife ulm study. J Gerontol B Psychol Sci Soc Sci. (2021) 76:1349–59. 10.1093/geronb/gbab023 33528511 PMC8363042

[60] Worrall C Jongenelis MI McEvoy PM Jackson B Newton RU Pettigrew S. An exploratory study of the relative effects of various protective factors on depressive symptoms among older people. Front Public Health. (2020) 8:579304. 10.3389/fpubh.2020.579304 33282813 PMC7690559

[61] Mock SE Eibach RP. Aging attitudes moderate the effect of subjective age on psychological well-being: evidence from a 10-year longitudinal study. Psychol Aging. (2011) 26:979–86. 10.1037/a0023877 21728444

[62] Rothermund K de Paula Couto MCP Fung HH Graf S Hess TM Liou S. Age-related attributions of experienced changes in life: origins and implications. J Gerontol B. (2021) 76:881–93. 10.1093/geronb/gbaa160 32909615

[63] Bodner E Ayalon L Avidor S Palgi Y. Accelerated increase and relative decrease in subjective age and changes in attitudes toward own aging over a 4-year period: results from the health and retirement study. Eur J Ageing. (2017) 14:17–27. 10.1007/s10433-016-0383-2 28804391 PMC5550619

[64] Cialani C Mortazavi R. The effect of objective income and perceived economic resources on self-rated health. Int J Equity Health. (2020) 19:196. 10.1186/s12939-020-01304-2 33148286 PMC7640443

[65] Knöchelmann A Seifert N Günther S Moor I Richter M. Income and housing satisfaction and their association with self-rated health in different life stages. a fixed effects analysis using a german panel study. BMJ Open. (2020) 10:e034294-e. 10.1136/bmjopen-2019-034294 32503868 PMC7279665

