# Supplementary material for: What Determines That Older Adults Feel Younger Than They Are? Results From a Nationally Representative Study in Germany
Source: Front Med (Lausanne). 2022 Jun 28;9:901420. doi: 10.3389/fmed.2022.901420 (PMC9274253; doi:10.3389/fmed.2022.901420)
Supplement: Supplementary file 1 [file Data_Sheet_1.ZIP › frontiers_fy_supp3.0.docx]

Supplementary Material

# Supplementary Tables

**Table S1:** Correlations of feeling younger.

|  | Feeling_younger |
| --- | --- |
| Age | 0.058*** |
| Sex | -0.016 |
| Retirement | 0.055*** |
| Residence | 0.023 |
| Education, low | -0.014 |
| Education, medium | -0.051** |
| Education, high | 0.057*** |
| Partner | 0.020 |
| Living_partner | 0.039* |
| Age_partner | -0.009 |
| Household | 0.001 |
| Children | 0.017 |
| Living_children | -0.034* |
| Relationship_partner | -0.051** |
| Relationship_family | -0.099*** |
| Relationship_friends | -0.098*** |
| Neighbors | -0.055*** |
| Sports | -0.153*** |
| Walks | -0.074*** |
| State_of_health | -0.295*** |
| Standard_of_living | -0.178*** |
| Corona_infection | 0.006 |
| Corona_environment | -0.020 |
| Corona_threat | -0.110*** |
| Depressiv | -0.247*** |
| Loneliness | -0.100*** |
| Life_satisfaction | 0.246*** |
| Attitudes | 0.312*** |

Values indicate the Spearman correlation coefficient r_s_ between feeling_younger and the considered variables of the total study population (**p<*0.05; ***p<*0.01; *** *p*<0.001).

**Table S2:** Network analysis, centrality measures.

| Variable | Betweenness | Closeness | Strength |
| --- | --- | --- | --- |
| Feeling_younger, FEY | 0.000 | 0.744 | 0.377 |
| Age, AGE | 0.973 | 0.952 | 0.934 |
| Sex, SEX | 0.411 | 0.941 | 0.678 |
| Retirement, RET | 0.000 | 0.874 | 0.590 |
| Residence, RES | 0.137 | 0.730 | 0.271 |
| Education, EDU | 0.521 | 0.960 | 0.432 |
| Partner, PAR | 0.000 | 0.834 | 0.565 |
| Living_partner, LIP | 0.356 | 0.895 | 0.682 |
| Age_partner, AGP | 0.466 | 0.968 | 0.855 |
| Household, HOU | 0.397 | 0.856 | 0.705 |
| Children, CHI | 0.000 | 0.532 | 0.108 |
| Living_children, LIC | 0.493 | 0.924 | 1.000 |
| Relationship_partner, RPA | 0.562 | 0.974 | 0.879 |
| Relationship_family, RFA | 0.123 | 0.743 | 0.479 |
| Relationship_friends, RFR | 0.164 | 0.768 | 0.571 |
| Neighbors, NEI | 0.096 | 0.683 | 0.271 |
| Sports, SPO | 0.342 | 0.842 | 0.491 |
| Walks, WAL | 0.000 | 0.723 | 0.228 |
| State_of_health, SOH | 1.000 | 0.976 | 0.761 |
| Standard_of_living, SOL | 0.616 | 1.000 | 0.599 |
| Corona_infection, COI | 0.041 | 0.485 | 0.260 |
| Corona_environment, COE | 0.288 | 0.519 | 0.312 |
| Corona_threat, COT | 0.000 | 0.722 | 0.292 |
| Depressiv, DEP | 0.411 | 0.893 | 0.595 |
| Loneliness, LON | 0.370 | 0.865 | 0.380 |
| Life_satisfaction, LSA | 0.068 | 0.848 | 0.476 |
| Attitudes, ATT | 0.096 | 0.897 | 0.558 |

The values for betweenness, closeness, and strength are given as relative values ranging from zero to one. A higher centrality measure indicates that a node is more central to the network.

**Table S3:** Network analysis, edge weights matrix.

| Variable | FEY | AGE | SEX | RET | RES | EDU | PAR | LIP | AGP | HOU | CHI | LIC | RPA | RFA |
| --- | --- | --- | --- | --- | --- | --- | --- | --- | --- | --- | --- | --- | --- | --- |
| FEY | 0.000 | 0.070 | 0.014 | 0.000 | 0.000 | 0.000 | 0.005 | 0.000 | 0.000 | 0.000 | 0.000 | 0.000 | 0.000 | 0.019 |
| AGE | 0.070 | 0.000 | 0.054 | 0.613 | 0.000 | 0.003 | 0.000 | 0.071 | 0.074 | 0.081 | 0.041 | 0.072 | 0.000 | 0.043 |
| SEX | 0.014 | 0.054 | 0.000 | 0.000 | 0.024 | 0.167 | 0.106 | 0.000 | 0.311 | 0.031 | 0.000 | 0.075 | 0.217 | 0.036 |
| RET | 0.000 | 0.613 | 0.000 | 0.000 | 0.026 | 0.036 | 0.026 | 0.021 | 0.024 | 0.036 | 0.000 | 0.042 | 0.000 | 0.000 |
| RES | 0.000 | 0.000 | 0.024 | 0.026 | 0.000 | 0.126 | 0.000 | 0.000 | 0.000 | 0.005 | 0.000 | 0.000 | 0.006 | 0.018 |
| EDU | 0.000 | 0.003 | 0.167 | 0.036 | 0.126 | 0.000 | 0.000 | 0.000 | 0.019 | 0.030 | 0.010 | 0.000 | 0.005 | 0.003 |
| PAR | 0.005 | 0.000 | 0.106 | 0.026 | 0.000 | 0.000 | 0.000 | 0.004 | 0.225 | 0.219 | 0.007 | 0.137 | 0.149 | 0.019 |
| LIP | 0.000 | 0.071 | 0.000 | 0.021 | 0.000 | 0.000 | 0.004 | 0.000 | 0.036 | 0.334 | 0.000 | 0.648 | 0.054 | 0.000 |
| AGP | 0.000 | 0.074 | 0.311 | 0.024 | 0.000 | 0.019 | 0.225 | 0.036 | 0.000 | 0.069 | 0.000 | 0.245 | 0.401 | 0.027 |
| HOU | 0.000 | 0.081 | 0.031 | 0.036 | 0.005 | 0.030 | 0.219 | 0.334 | 0.069 | 0.000 | 0.078 | 0.184 | 0.046 | 0.000 |
| CHI | 0.000 | 0.041 | 0.000 | 0.000 | 0.000 | 0.010 | 0.007 | 0.000 | 0.000 | 0.078 | 0.000 | 0.000 | 0.000 | 0.025 |
| LIC | 0.000 | 0.072 | 0.075 | 0.042 | 0.000 | 0.000 | 0.137 | 0.648 | 0.245 | 0.184 | 0.000 | 0.000 | 0.269 | 0.002 |
| RPA | 0.000 | 0.000 | 0.217 | 0.000 | 0.006 | 0.005 | 0.149 | 0.054 | 0.401 | 0.046 | 0.000 | 0.269 | 0.000 | 0.074 |
| RFA | 0.019 | 0.043 | 0.036 | 0.000 | 0.018 | 0.003 | 0.019 | 0.000 | 0.027 | 0.000 | 0.025 | 0.002 | 0.074 | 0.000 |
| RFR | 0.000 | 0.005 | 0.027 | 0.003 | 0.023 | 0.020 | 0.004 | 0.000 | 0.022 | 0.000 | 0.007 | 0.010 | 0.007 | 0.272 |
| NEI | 0.000 | 0.000 | 0.000 | 0.002 | 0.006 | 0.000 | 0.000 | 0.000 | 0.000 | 0.061 | 0.000 | 0.000 | 0.012 | 0.061 |
| SPO | 0.039 | 0.033 | 0.017 | 0.019 | 0.051 | 0.123 | 0.000 | 0.000 | 0.001 | 0.000 | 0.000 | 0.005 | 0.000 | 0.000 |
| WAL | 0.020 | 0.020 | 0.026 | 0.000 | 0.000 | 0.000 | 0.001 | 0.000 | 0.000 | 0.000 | 0.000 | 0.000 | 0.000 | 0.000 |
| SOH | 0.139 | 0.097 | 0.000 | 0.041 | 0.030 | 0.012 | 0.006 | 0.000 | 0.000 | 0.001 | 0.000 | 0.007 | 0.008 | 0.016 |
| SOL | 0.047 | 0.028 | 0.008 | 0.015 | 0.078 | 0.143 | 0.009 | 0.013 | 0.009 | 0.001 | 0.000 | 0.000 | 0.077 | 0.047 |
| COI | 0.000 | 0.000 | 0.007 | 0.015 | 0.000 | 0.000 | 0.005 | 0.000 | 0.020 | 0.001 | 0.000 | 0.000 | 0.000 | 0.013 |
| COE | 0.009 | 0.048 | 0.004 | 0.028 | 0.053 | 0.012 | 0.000 | 0.000 | 0.000 | 0.000 | 0.000 | 0.009 | 0.000 | 0.000 |
| COT | 0.008 | 0.037 | 0.011 | 0.000 | 0.024 | 0.000 | 0.013 | 0.000 | 0.000 | 0.000 | 0.018 | 0.027 | 0.000 | 0.010 |
| DEP | 0.071 | 0.029 | 0.033 | 0.009 | 0.000 | 0.036 | 0.016 | 0.000 | 0.000 | 0.006 | 0.000 | 0.000 | 0.046 | 0.007 |
| LON | 0.002 | 0.000 | 0.000 | 0.000 | 0.000 | 0.003 | 0.030 | 0.000 | 0.000 | 0.008 | 0.000 | 0.000 | 0.077 | 0.088 |
| LSA | 0.071 | 0.080 | 0.008 | 0.000 | 0.000 | 0.000 | 0.000 | 0.000 | 0.000 | 0.029 | 0.000 | 0.000 | 0.050 | 0.040 |
| ATT | 0.138 | 0.120 | 0.000 | 0.069 | 0.000 | 0.000 | 0.000 | 0.000 | 0.000 | 0.003 | 0.000 | 0.000 | 0.024 | 0.009 |

The values indicate the edge weights, which describe the strength of the connection between two nodes. Values are ranging from zero to one. A higher edge weight indicates that the connection between the two nodes is stronger. Abbreviations in the order of their appearance: FEY, *feeling_younger*; AGE, *age*; SEX, *sex*; RET, *retirement*; RES, *residence*; EDU, *education*; PAR, *partner*; LIP, *living_partner*; AGP, *age_partner*; HOU, *household*; CHI, *children*; LIC, *living_children*; RPA, *relationship_partner*; RFA, *relationship_family*; RFR, *relationship_friends*; NEI, *neighbors*; SPO, *sports*; WAL, *walks*; SOH, *state_of_health*; SOL, *standard_of_living*; COI, *corona_infection*; COE, *corona_environment*; COT, *corona_threat*; DEP, *depressive*; LON, *loneliness*; LSA, *life_satisfaction*; ATT, *attitudes*.

**Table S3:** Network analysis, edge weights matrix, continued.

| Variable | RFR | NEI | SPO | WAL | SOH | SOL | COI | COE | COT | DEP | LON | LSA | ATT |
| --- | --- | --- | --- | --- | --- | --- | --- | --- | --- | --- | --- | --- | --- |
| FEY | 0.000 | 0.000 | 0.039 | 0.020 | 0.139 | 0.047 | 0.000 | 0.009 | 0.008 | 0.071 | 0.002 | 0.071 | 0.138 |
| AGE | 0.005 | 0.000 | 0.033 | 0.020 | 0.097 | 0.028 | 0.000 | 0.048 | 0.037 | 0.029 | 0.000 | 0.080 | 0.120 |
| SEX | 0.027 | 0.000 | 0.017 | 0.026 | 0.000 | 0.008 | 0.007 | 0.004 | 0.011 | 0.033 | 0.000 | 0.008 | 0.000 |
| RET | 0.003 | 0.002 | 0.019 | 0.000 | 0.041 | 0.015 | 0.015 | 0.028 | 0.000 | 0.009 | 0.000 | 0.000 | 0.069 |
| RES | 0.023 | 0.006 | 0.051 | 0.000 | 0.030 | 0.078 | 0.000 | 0.053 | 0.024 | 0.000 | 0.000 | 0.000 | 0.000 |
| EDU | 0.020 | 0.000 | 0.123 | 0.000 | 0.012 | 0.143 | 0.000 | 0.012 | 0.000 | 0.036 | 0.003 | 0.000 | 0.000 |
| PAR | 0.004 | 0.000 | 0.000 | 0.001 | 0.006 | 0.009 | 0.005 | 0.000 | 0.013 | 0.016 | 0.030 | 0.000 | 0.000 |
| LIP | 0.000 | 0.000 | 0.000 | 0.000 | 0.000 | 0.013 | 0.000 | 0.000 | 0.000 | 0.000 | 0.000 | 0.000 | 0.000 |
| AGP | 0.022 | 0.000 | 0.001 | 0.000 | 0.000 | 0.009 | 0.020 | 0.000 | 0.000 | 0.000 | 0.000 | 0.000 | 0.000 |
| HOU | 0.000 | 0.061 | 0.000 | 0.000 | 0.001 | 0.001 | 0.001 | 0.000 | 0.000 | 0.006 | 0.008 | 0.029 | 0.003 |
| CHI | 0.007 | 0.000 | 0.000 | 0.000 | 0.000 | 0.000 | 0.000 | 0.000 | 0.018 | 0.000 | 0.000 | 0.000 | 0.000 |
| LIC | 0.010 | 0.000 | 0.005 | 0.000 | 0.007 | 0.000 | 0.000 | 0.009 | 0.027 | 0.000 | 0.000 | 0.000 | 0.000 |
| RPA | 0.007 | 0.012 | 0.000 | 0.000 | 0.008 | 0.077 | 0.000 | 0.000 | 0.000 | 0.046 | 0.077 | 0.050 | 0.024 |
| RFA | 0.272 | 0.061 | 0.000 | 0.000 | 0.016 | 0.047 | 0.013 | 0.000 | 0.010 | 0.007 | 0.088 | 0.040 | 0.009 |
| RFR | 0.000 | 0.154 | 0.016 | 0.014 | 0.031 | 0.075 | 0.006 | 0.018 | 0.027 | 0.059 | 0.116 | 0.028 | 0.045 |
| NEI | 0.154 | 0.000 | 0.024 | 0.000 | 0.004 | 0.000 | 0.035 | 0.000 | 0.004 | 0.000 | 0.069 | 0.005 | 0.032 |
| SPO | 0.016 | 0.024 | 0.000 | 0.293 | 0.132 | 0.034 | 0.000 | 0.000 | 0.000 | 0.063 | 0.000 | 0.000 | 0.004 |
| WAL | 0.014 | 0.000 | 0.293 | 0.000 | 0.021 | 0.000 | 0.000 | 0.000 | 0.000 | 0.000 | 0.000 | 0.000 | 0.000 |
| SOH | 0.031 | 0.004 | 0.132 | 0.021 | 0.000 | 0.148 | 0.000 | 0.007 | 0.158 | 0.227 | 0.000 | 0.049 | 0.183 |
| SOL | 0.075 | 0.000 | 0.034 | 0.000 | 0.148 | 0.000 | 0.018 | 0.002 | 0.044 | 0.031 | 0.014 | 0.193 | 0.005 |
| COI | 0.006 | 0.035 | 0.000 | 0.000 | 0.000 | 0.018 | 0.000 | 0.318 | 0.000 | 0.005 | 0.000 | 0.008 | 0.000 |
| COE | 0.018 | 0.000 | 0.000 | 0.000 | 0.007 | 0.002 | 0.318 | 0.000 | 0.027 | 0.007 | 0.000 | 0.000 | 0.000 |
| COT | 0.027 | 0.004 | 0.000 | 0.000 | 0.158 | 0.044 | 0.000 | 0.027 | 0.000 | 0.036 | 0.000 | 0.004 | 0.057 |
| DEP | 0.059 | 0.000 | 0.063 | 0.000 | 0.227 | 0.031 | 0.005 | 0.007 | 0.036 | 0.000 | 0.200 | 0.043 | 0.108 |
| LON | 0.116 | 0.069 | 0.000 | 0.000 | 0.000 | 0.014 | 0.000 | 0.000 | 0.000 | 0.200 | 0.000 | 0.049 | 0.002 |
| LSA | 0.028 | 0.005 | 0.000 | 0.000 | 0.049 | 0.193 | 0.008 | 0.000 | 0.004 | 0.043 | 0.049 | 0.000 | 0.168 |
| ATT | 0.045 | 0.032 | 0.004 | 0.000 | 0.183 | 0.005 | 0.000 | 0.000 | 0.057 | 0.108 | 0.002 | 0.168 | 0.000 |

The values indicate the edge weights, which describe the strength of the connection between two nodes. Values are ranging from zero to one. A higher edge weight indicates that the connection between the two nodes is stronger. Abbreviations in the order of their appearance: FEY, *feeling_younger*; AGE, *age*; SEX, *sex*; RET, *retirement*; RES, *residence*; EDU, *education*; PAR, *partner*; LIP, *living_partner*; AGP, *age_partner*; HOU, *household*; CHI, *children*; LIC, *living_children*; RPA, *relationship_partner*; RFA, *relationship_family*; RFR, *relationship_friends*; NEI, *neighbors*; SPO, *sports*; WAL, *walks*; SOH, *state_of_health*; SOL, *standard_of_living*; COI, *corona_infection*; COE, *corona_environment*; COT, *corona_threat*; DEP, *depressive*; LON, *loneliness*; LSA, *life_satisfaction*; ATT, *attitudes*.

**Table S4:** Regression analysis, variables in the equation.

|  |  | B | p | Exp(B) | 95% CI lb | 95% CI ub |
| --- | --- | --- | --- | --- | --- | --- |
| Step 1 | age | 0.023 | 0.029 | 1.023 | 1.002 | 1.045 |
|  | sex | 0.304 | 0.073 | 1.355 | 0.972 | 1.891 |
|  | retirement | -0.408 | 0.049 | 0.665 | 0.442 | 0.998 |
|  | residence | -0.079 | 0.596 | 0.924 | 0.691 | 1.237 |
|  | education_1 | 0.137 | 0.689 | 1.147 | 0.586 | 2.245 |
|  | education_3 | -0.170 | 0.241 | 0.844 | 0.635 | 1.121 |
|  | living_partner | 19.032 | 0.998 | 184342458.377 | 0.000 | . |
|  | age_partner | -0.112 | 0.495 | 0.894 | 0.648 | 1.234 |
|  | children | 0.035 | 0.577 | 1.036 | 0.915 | 1.172 |
|  | living_children | 0.048 | 0.805 | 1.049 | 0.716 | 1.539 |
|  | relationship_partner | 0.164 | 0.103 | 1.178 | 0.968 | 1.434 |
|  | relationship_family | -0.170 | 0.092 | 0.844 | 0.692 | 1.028 |
|  | relationship_friends | 0.151 | 0.195 | 1.163 | 0.926 | 1.462 |
|  | neighbors | -0.050 | 0.581 | 0.951 | 0.796 | 1.136 |
|  | sports | -0.096 | 0.046 | 0.909 | 0.827 | 0.998 |
|  | walks | 0.025 | 0.603 | 1.026 | 0.932 | 1.129 |
|  | state_of_health | -0.625 | <.001 | 0.535 | 0.432 | 0.662 |
|  | standard_of_living | -0.185 | 0.059 | 0.831 | 0.686 | 1.007 |
|  | corona_infection | -0.747 | 0.507 | 0.474 | 0.052 | 4.313 |
|  | corona_environment | 0.215 | 0.389 | 1.240 | 0.760 | 2.021 |
|  | corona_threat | -0.053 | 0.084 | 0.948 | 0.892 | 1.007 |
|  | depressive | 0.468 | 0.004 | 1.597 | 1.159 | 2.199 |
|  | loneliness | -0.118 | 0.604 | 0.889 | 0.570 | 1.386 |
|  | life_satisfaction | -0.491 | 0.011 | 0.612 | 0.420 | 0.893 |
|  | attitudes | -1.023 | <.001 | 0.360 | 0.258 | 0.502 |
| Step 15 | age | 0.025 | 0.012 | 1.025 | 1.006 | 1.046 |
|  | retirement | -0.389 | 0.056 | 0.678 | 0.454 | 1.010 |
|  | living_partner | 19.060 | 0.998 | 189571079.934 | 0.000 | . |
|  | relationship_partner | 0.175 | 0.066 | 1.191 | 0.989 | 1.435 |
|  | sports | -0.097 | 0.026 | 0.908 | 0.833 | 0.989 |
|  | state_of_health | -0.630 | <.001 | 0.533 | 0.432 | 0.658 |
|  | standard_of_living | -0.209 | 0.026 | 0.811 | 0.675 | 0.975 |
|  | corona_threat | -0.054 | 0.075 | 0.947 | 0.892 | 1.005 |
|  | depressive | 0.436 | 0.007 | 1.546 | 1.128 | 2.120 |
|  | life_satisfaction | -0.459 | 0.016 | 0.632 | 0.435 | 0.918 |
|  | attitudes | -1.022 | <.001 | 0.360 | 0.259 | 0.501 |

Values were obtained using binomial logistic regression analysis with backward selection (likelihood ratio) to identify predictors of feeling younger. CI lb: Lower bound of the confidence interval. CI ub: upper bound of the confidence interval. Exp(B): Standardized regression coefficient. B: Unstandardized regression coefficient.

# Supplementary Figures

| 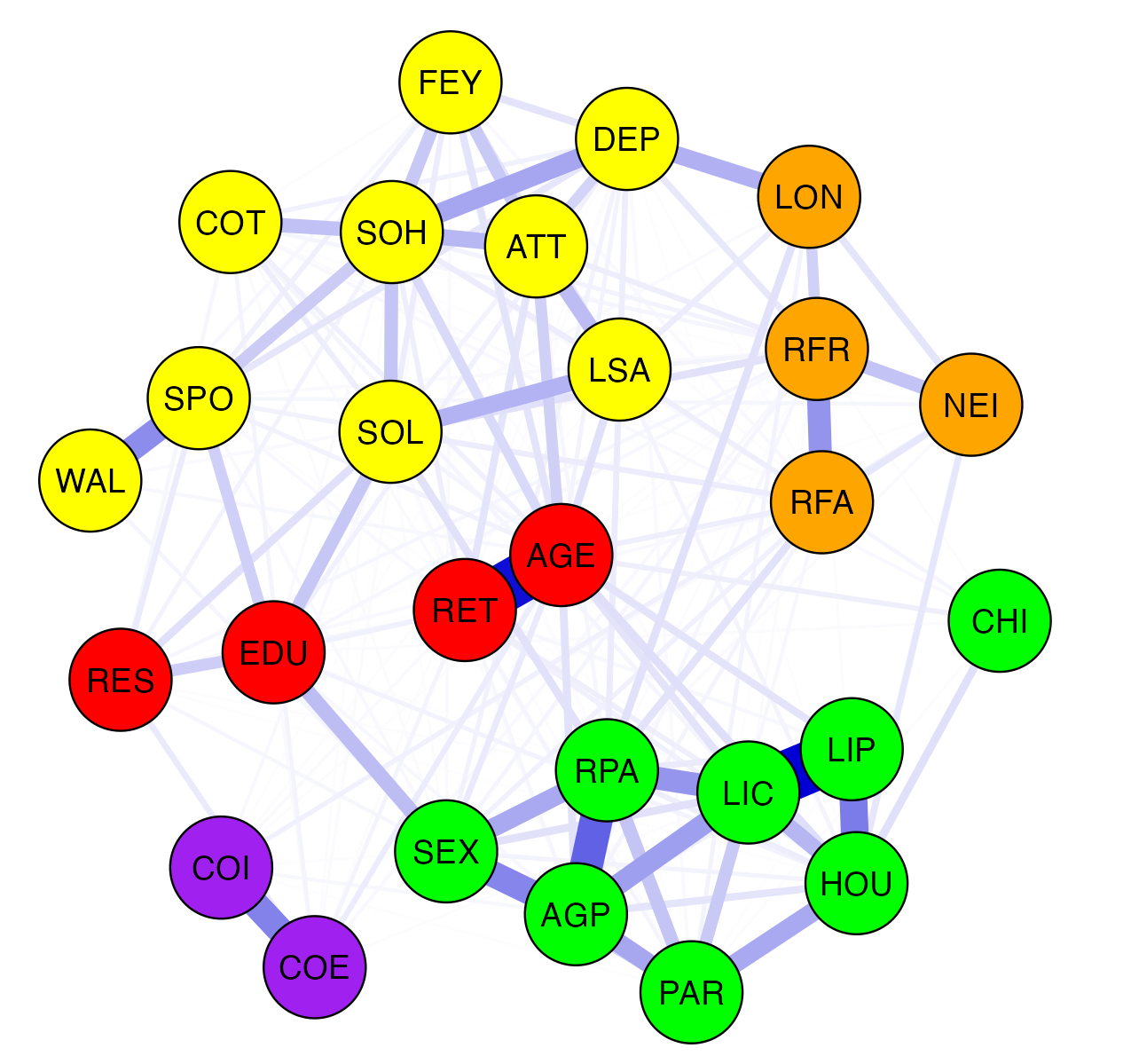 | FEY, Feeling_younger  AGE, Age  SEX, Sex  RET, Retirement  RES, Residence  EDU, Education  PAR, Partner  LIP, Living_partner  AGP, Age_partner  HOU, Household  CHI, Children  LIC, Living_children  RPA, Relationship_partner  RFA, Relationship_family  RFR, Relationship_friends  NEI, Neighbors  SPO, Sports  WAL, Walks  SOH, State_of_health  SOL, Standard_of_living  COI, Corona_infection  COE, Corona_environment  COT, Corona_threat  DEP, Depressive  LON, Loneliness  LSA, Life_satisfaction  ATT, Attitudes |
| --- | --- |

**Figure S1.** Domain network structure of the total study population. The nodes display the variables, and the edges represent correlations between the nodes. The thickness of the edges corresponds to the strength of the correlation. Green: items describing the household composition (*sex*, SEX; *partner*, PAR; *age_partner*, AGP; *relationship_partner*, RPA; *living_partner*, LIP; *living_children*, LIC; *children*, CHI; *household*, HOU). Orange: items describing social contacts (*loneliness*, LON; *relationship_family*, RFA; *relationship_friends*, RFR; *neighbors*, NEI). Yellow: items describing wellbeing (*feeling_younger*, FEY; *state_of_health*, SOH; *attitudes*, ATT; *depressive*, DEP; *corona_threat*, COT; *standard_of_living*, SOL; *life_satisfaction*, LSA; *sports*, SPO; *walks*, WAL). Purple: items describing a corona infection (*corona_infection*, COI; *corona_environment*, COE). Red: items that cannot be categorized visually into a domain (*age*, AGE; *retirement*, RET; *education*, EDU; *residence*, RES).

**
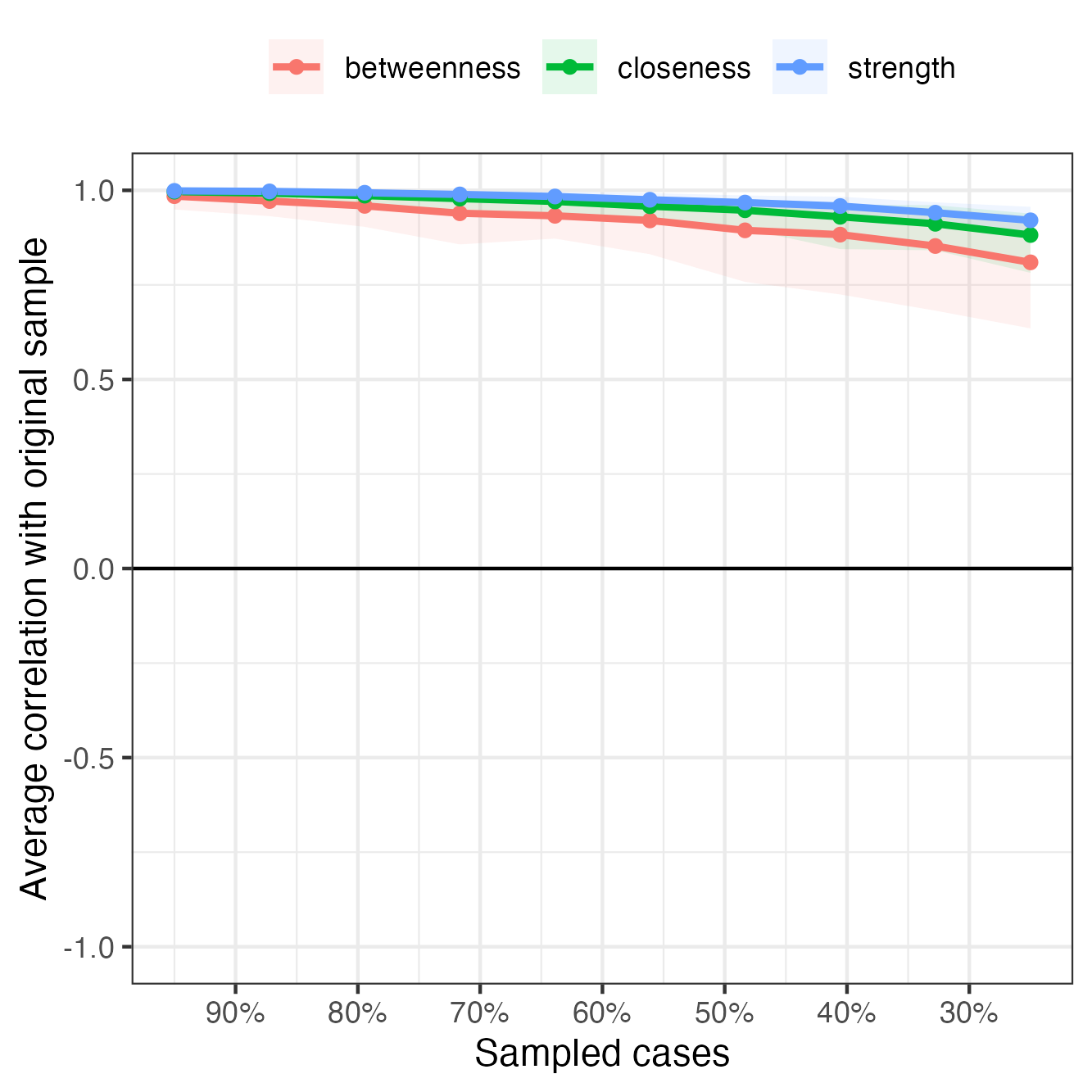
**

**Figure S2:** Case-dropping bootstrapped procedure of centrality indices (number of bootstraps = 1,000). The correlations of the centrality measures between the original sample and those from the subsamples with an increasingly higher percentage of dropped-out cases were calculated. The correlation stability coefficient (*CS coefficient*) represents the maximum proportion of cases which can be dropped to maintain a correlation of at least 0.70 with the original centrality indices. The 95% confidence interval of these correlations are colored. The case-dropping bootstrap procedure shows that *CS coefficient* of node betweenness (CS (cor=0.7) = 0.66), closeness (CS (cor=0.7) > 0.75), and strength (CS (cor=0.7) > 0.75) remained highly stable.

**
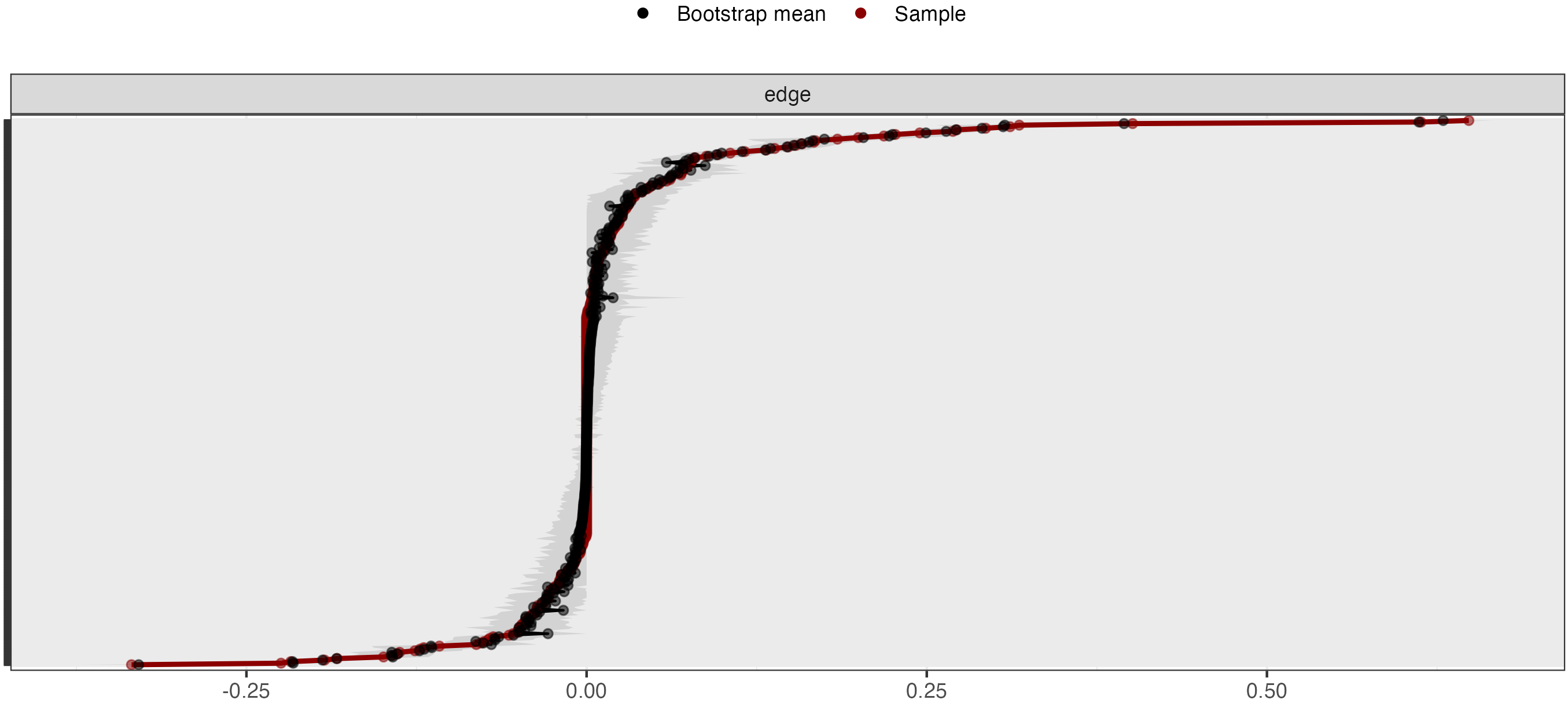
**

**Figure S3:** Nonparametric bootstrapped procedure of edge weights (number of bootstraps = 1,000). Using a nonparametric bootstrapped procedure, the 95% bootstrapped confidence interval of edge weights were determined around the regularized edge weights. Each horizontal line represents an edge. The labeling of the y-axis was omitted to avoid overlapping. The red dots represent the original sample values. The black dots represent the bootstrap values. The grey areas represent the 95% bootstrapped confidence intervals. The bootstrapped confidence intervals are narrow, suggesting accurate results.

**
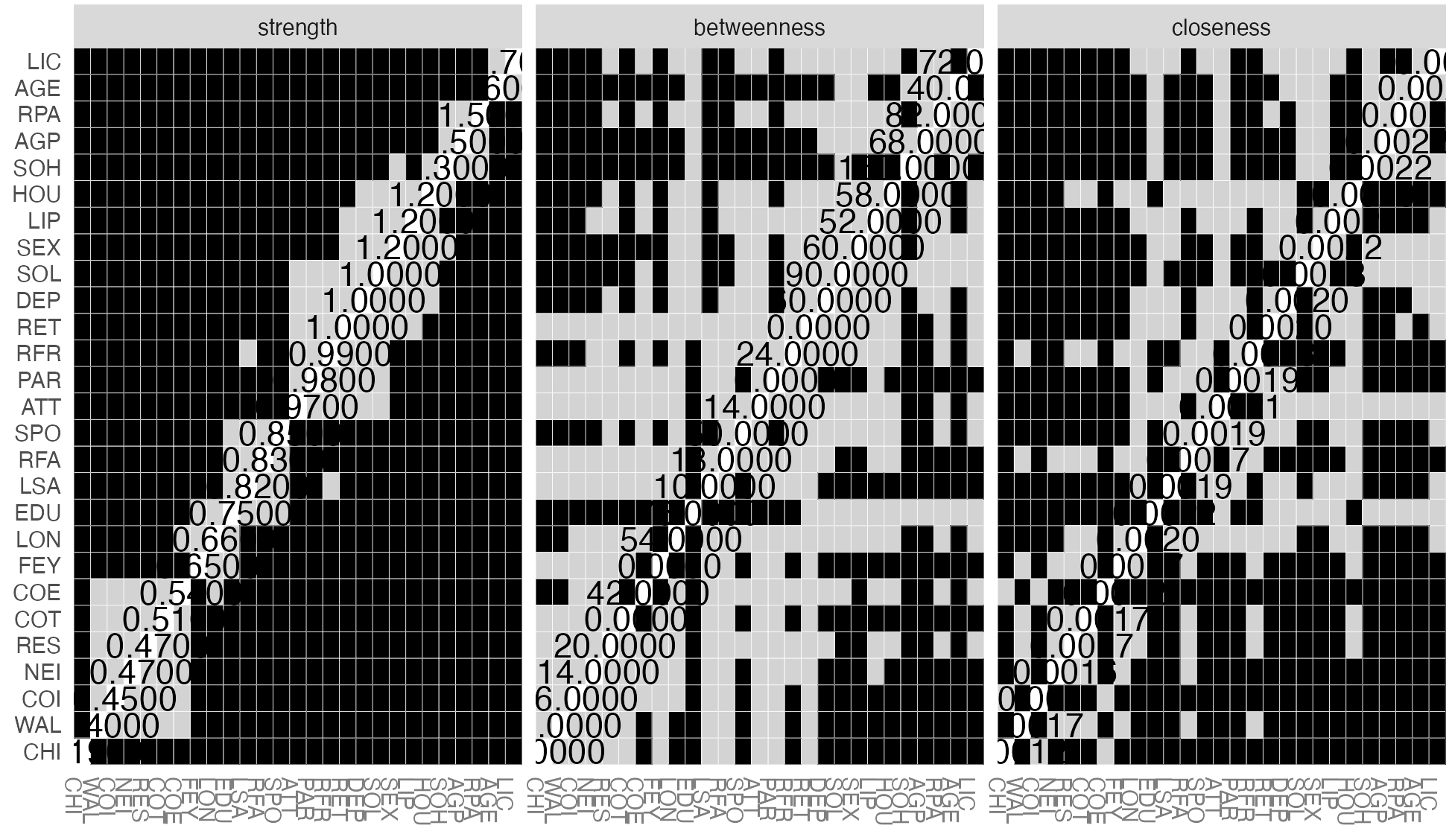
**

**Figure S4:** Nonparametric bootstrapped centrality difference test (number of bootstraps = 1,000). A bootstrapped difference test (α= 0.05) was used to determine if the nodes were significantly different from each other regarding centrality measures. Each point on the x and y axes represents a network node. Gray boxes indicate that two nodes do not significantly differ from each other. Black boxes indicate that two nodes significantly differ from each other. The numbers in the diagonal represent the value of the centrality measure of the node.
